# Soluble Sema4D in Plasma of Head and Neck Squamous Cell Carcinoma Patients Is Associated With Underlying Non-Inflamed Tumor Profile

**DOI:** 10.3389/fimmu.2021.596646

**Published:** 2021-03-11

**Authors:** Rania H. Younis, Ioana Ghita, Manar Elnaggar, Risa Chaisuparat, Vasileios Ionas Theofilou, Donita Dyalram, Robert A. Ord, Eduardo Davila, Luke J. Tallon, John C. Papadimitriou, Tonya J. Webb, Søren M. Bentzen, Joshua E. Lubek

**Affiliations:** ^1^ Department of Oncology and Diagnostic Sciences, University of Maryland School of Dentistry, Baltimore, MD, United States; ^2^ Tumor Immunology and Immunotherapy Division, University of Maryland Marlene and Stewart Greenebaum Comprehensive Cancer Center, Baltimore, MD, United States; ^3^ Department of Oral Pathology, Faculty of Dentistry, University of Alexandria, Alexandria, Egypt; ^4^ Department of Oral Pathology, Faculty of Dentistry, Chulalongkorn University, Bangkok, Thailand; ^5^ Department of Oral Medicine and Pathology, School of Dentistry, National and Kapodistrian University of Athens, Athens, Greece; ^6^ Department of Oral and Maxillofacial Surgery, University of Maryland School of Dentistry, Baltimore, MD, United States; ^7^ Department of Medicine, University of Colorado, Aurora, CO, United States; ^8^ The Institute for Genome Sciences, University of Maryland School of Medicine, Baltimore, MD, United States; ^9^ Department of Pathology, University of Maryland School of Medicine, Baltimore, MD, United States; ^10^ Department of Microbiology and Immunology, University of Maryland School of Medicine, Baltimore, MD, United States; ^11^ Division of Biostatistics and Bioinformatics, Department of Epidemiology and Public Health, University of Maryland School of Medicine, Baltimore, MD, United States

**Keywords:** soluble, head and neck squamous cell carcinoma (HNSCC)****, Sema4D, immune excluded, real time, IFN-*γ*, biomarker, non-inflamed

## Abstract

Semaphorin 4D (Sema4D) is a glycoprotein that is expressed by several tumors and immune cells. It can function as a membrane bound protein or as a cleaved soluble protein (sSema4D). We sought to investigate the translational potential of plasma sSema4D as an immune marker in plasma of patients with head and neck squamous cell carcinoma (HNSCC). Paired peripheral blood and tumor tissue samples of 104 patients with HNSCC were collected at the same time point to allow for real time analysis. Scoring of the histological inflammatory subtype (HIS) was carried out using Sema4D immunohistochemistry on the tumor tissue. sSema4D was detected in plasma using direct ELISA assay. Defining elevated sSema4D as values above the 95^th^ percentile in healthy controls, our data showed that sSema4D levels in plasma were elevated in 25.0% (95% CI, 16.7–34.9%) of the patients with HNSCC and showed significant association with HIS immune excluded (HIS-IE) (p = 0.007), Sema4D^+ve^ tumor cells (TCs) (p = 0.018) and PD-L1^+ve^ immune cells (ICs) (p = 0.038). A multi-variable logistic regression analysis showed that HIS was significantly (P = 0.004) associated with elevated sSema4D, an association not explained by available patient-level factors. Using the IO-360 nanoString platform, differential gene expression (DGE) analysis of 10 HNSCC tumor tissues showed that patients with high sSema4D in plasma (HsS4D) clustered as IFN-*γ* negative tumor immune signature and were mostly HIS-IE. The IC type in the HsS4D paired tumor tissue was predominantly myeloid, while the lymphoid compartment was higher in the low sSema4D (LsS4D). The Wnt signaling pathway was upregulated in the HsS4D group. Further analysis using the IO-360, 770 gene set, showed significant non-inflamed profile of the HsS4D tumors compared to the LsS4D. In conclusion, our data reveals an association between sSema4D and the histological inflammatory subtype.

## Introduction

Head and neck squamous cell carcinoma (HNSCC) is a devastating malignancy that occurs in close proximity to vital structures. A projection for the year 2020 estimated that 53,260 new cases, and 10,750 annual deaths of oral and pharyngeal SCC will occur in the US (1). Surgical excision remains the first line of treatment for oral cavity cancer. Depending on the disease presentation and pathological findings, other therapies are often required including radiotherapy as a single or adjuvant option, chemotherapy, targeted agents and immunotherapy as the most recent therapeutic advent (2). The overall 5 year survival rate is 65% with an average 6–10 month survival rate for platinum resistant patients (3). Several studies have described HNSCC as an immune suppressive tumor (4, 5). The recent advent of immunotherapy showed unprecedented improvement in overall response of advanced stage malignancy (2). Further understanding of HNSCC tumor inflammation can provide the basis for tumor stratification according to the underlying immune profile and hence may result in better treatment strategies and patient outcomes (6).

Immunoscore revolutionized our understanding of the histological patterns of HNSCC inflammation (7, 8). Initially referring to the inflamed tumor cores as hot and the non-inflamed as altered, the later descriptions included the immune excluded and the immune desert as the cold subtype (9, 10). The cold subtype showed poor prognosis with higher recurrence rate (9, 11). In addition, evidence-using the combination of inflammation and tumor mutational burden analysis revealed other intermediate subtypes that can include low mutational burden tumors that can be inflamed but with rich fibroblastic signature (12). In concordance, the inflamed tumor surrogate biomarker, programmed death ligand 1 (PD-L1) is used as a single analyte (13, 14) or the interferon gamma (IFN-γ) tumor immune signature as a multi-analyte, to represent T cell inflamed or non-inflamed tumors (15). IFN-*γ* plays a key role in antitumor response. It is produced by activated T cells, as well as natural killer (NK) and NKT cells. The IFN-*γ* induced gene response includes pro-inflammatory, as well as feedback inhibitory signals, a feature that tumors take advantage of to progress and advance. An expanded and refined IFN-*γ* immune signature was initially generated using the nanostring nCounter IO-360 platform on melanoma samples, then tested on HNSCC and gastric cancer (15). The differential gene expression (DGE) of the IFN-*γ* signature was significantly discriminatory of the HNSCC response to anti-PD-1/PD-L1 standard immunotherapy. The final IFN-*γ* signature that further confirmed the significant correlation with HNSCC response to pembrolizumab from tumor samples obtained from KEYNOTE-012 was also validated on nine types of malignancies (13). It included 18 functional genes, that encompassed pro-inflammatory cytokines/chemokines (CXCR6, CCL5, CXCL9, STAT1, CMKLR1), T cell markers (CD8A, CD27,TIGIT), NK cell activity (NKG7, HLA-E), antigen presentation (HLA-DRB1, HLA-DQA1, PSMB10), and additional immunomodulatory factors (LAG3, IDO1, PDCD1LG2, CD274/PD-L1, CD276) (15). This immune signature was based on available tumor tissue biopsies. Tumor tissue represents an essential source of information that can be used in determining the course of treatment. Yet, in later stage malignancy, serial biopsies may not be feasible. Soluble immune biomarkers in the blood can potentially provide critical information at the initiation of immunotherapy or at any time point of the patient care in response to treatment (16–19). However, impact of soluble immune biomarkers in HNSCC is still limited, requiring further studies.

Semaphorin 4D (Sema4D; a.k.a. CD100) is an emerging immune biomarker that belongs to the fourth group of the Semaphorin family, which shares a conserved N-terminal domain called the ‘sema’ domain with highly conserved cysteine residues (20). Sema4D is expressed by almost all white blood cells of both lymphoid and myeloid origin (21–25). Interestingly, it can be expressed by activated T cells to bind its low affinity receptor CD72 on B cells or on antigen presenting cells, which results in the activation of humoral or T cell-mediated immunity, respectively (26, 27). Sema4D is a transmembrane glycoprotein that can function in the bound or soluble form. MT1-MMP and ADAM17 have been implicated in Sema4D proteolytic cleavage and shedding (28–30). The soluble form of Sema4D (sSema4D) inhibits myeloid cell spontaneous and cytokine-induced migration (22) (31). In the tumor microenvironment HNSCC-derived sSema4D was shown to induce immune suppression through upregulation of myeloid derived suppressor cells (MDSC) (5), as well as the increase of extracellular collagen deposition by fibroblasts (32). The expression of Sema4D has been described in tumor cells (TC) of several malignancies including HNSCC and by tumor infiltrating immune cells (IC), including tumor associated macrophages (TAMs) (32, 33). Sema4D also promotes tumor migration and invasion through Rho activation, microtubule organization, and epithelial mesenchymal transition (34). It has been associated with overall poor prognosis in sarcomas and cutaneous SCC (35, 36). Importantly, sSema4D is present in the peripheral blood of HNSCC patients and other pathologic conditions including heart failure, autoimmunity and allergy (37–39). These findings reflect the significance of Sema4D as a soluble immune biomarker, and we sought to investigate its translational potential in HNSCC.

In the present work, we used paired tumor tissue and plasma samples obtained at the same time point to allow for real time analysis. The cohort was mainly immunotherapy naïve patients with HNSCC treated with surgical excision as the initial line of treatment. The current data suggests the potential of sSema4D as a soluble immune biomarker that can read non-inflamed tumor HIS and low IFN-*γ* immune signature in real time. It highlights Sema4D as a target for inhibition to be further investigated as a method to sensitize HNSCC to immunotherapy.

## Materials and Methods

### Paired Tumor Tissue and Plasma of HNSCC Patients

Under an Institutional Review Board (IRB) approved protocol at the University of Maryland School of Medicine (UMSOM) (HP-00073603), paired blood samples and whole excision tumor tissue were collected prospectively at the day of surgery from 104 HNSCC upon informed patient consent. Our main inclusion criteria were primary tumors resected by surgery as the initial line of treatment. The blood was drawn pre-operatively prior to the planned surgical excision and processed to obtain plasma within 2 h of collection. Plasma from 51 patients with chronic pathological conditions served as controls. These included patients with autoimmune conditions (31 cases), allergy (10 cases) and osteoarthritis (10 cases) and 11 samples from healthy donors were collected retrospectively under an approved UMSOM IRB protocol HP-00074877. An additional 20 healthy donor plasma samples were purchased from Innovative Research (Novi, MI). All blood samples were processed to obtain plasma in sodium heparin tubes (BD Vacutainer glass tubes Medex Supply, NY; cat # 366480).

### Sema4D ELISA

Sema4D concentration in the plasma was determined using direct ELISA as previously described (5). Briefly, Immulon 4 HBX microtiter plates (Thermo Scientific, Waltham, MA) were coated with undiluted plasma, washed with ELISA washing buffer, then incubated with anti-human CD100 antibody (clone: 133-1C6; Invitrogen, eBioscience, CA; cat # 14-1009-82) overnight, then followed by Goat anti-mouse IgM-Heavy chain, HRP conjugate secondary antibody (Invitrogen USA, IL; cat. # 62-6820), detection with TMB (Biolegend, CA; cat #421101) and Stop Solution (Biolegend, CA; cat #77316). The concentrations of Sema4D were calculated using the standard curve established using recombinant Sema4D (catalog no. 310- 29) (Peprotech, RockyHill, NJ). The detection limit was 3.1–1000 ng/ml. Plates were read at 450 nm wavelength using BioTek Epoch microplate spectrophotometer.

### Immunohistochemistry

For Sema4D staining, the avidin–biotin complex (ABC) technique was used following Vectastain elite ABC kit (PK-6102, mouse IgG) (Vector Laboratories, CA). Briefly, FFPE tissue sections were deparaffinized, then rehydrated in graded ethanol, treated with Tris- EDTA buffer for antigen retrieval, and quenched in hydrogen peroxide to block endogenous peroxidase. Tissue sections were blocked with 2.5% normal plasma, incubated overnight at 4°C with anti- Sema4D antibody (clone 30/CD100; Catalog no. 610670) (BD Transduction Laboratories), followed by biotinylated secondary antibody (catalog no. BA-9200), then the ABC reagent. Primary antibody was omitted for negative control. Diaminobenzidine (SK-4105) was used as chromogen and counterstained with Mayer’s hematoxylin (Sigma-Aldrich Corp.). PD-L1 staining (clone 28-8, catalog no. ab205921, Abcam) was used to stain the HNSCC sections according to Abcam IHC protocol. Universal HIER antigen retrieval reagent catalog # (ab208572), Rabbit specific IHC polymer detection kit HRP/DAB catalog # (ab209101) Amplifier and Detector, and DAB substrate kit catalog # (ab64238) were used from Abcam according to their PD-L1 IHC protocol.

The Sema4D and PD-L1 labeling index (LI) reflecting the intensity and extent of staining in the TC and the IC was defined semi-quantitatively using intensity and percentage of staining as (0, 1, 2, and 3). (0) was negative, (1) focal or diffuse weak staining, (2) focal strong positivity ≤25%, and (3) for diffuse strong positivity >25%. The combined positive score (CPS) was counted for both TC and IC positivity. The immunohistochemical (IHC) score standardization was carried by the surgical pathologists (JP, RC, RY), then the 104 HNSCC samples were scored by (RY and RC). Discordant scores were adjudicated by JP. Slides were scanned using Leica biosystem scanscope. Histological features included extent of peritumoral stromal fibrosis, the extent of inflammation taking into consideration the size of the tumor islands in relation to the number of immune (IC) infiltrate. The Histological inflammatory subtype (HIS) was scored according to Sema4D positive IC infiltrate into the tumor core; IC excluded by thin peritumoral fibrous rim or only at the tumor margin, or no IC in stroma, was carried using the *Aperio Imagescope.*

### RNA Extraction and nSolver Analysis

For RNA extraction, three to five unstained, 5 mm thick FFPE tissue sections from 10 cases were used. Tumor tissue including 1–3 mm peritumoral stroma was mapped, manually micro-dissected by surgical oral pathologist (RY), and scrapped out the slides, guided by one H&E stained section of each tumor. RNA extraction using Rneasy FFPE kit (Qiagen, catalog no 73504) was carried out in the Genomic Core Facility, University of Maryland Baltimore. RNA quality control (QC) analysis was run on nanochip to ensure the required 200 nt in 50–300 ng with no prior amplification or enzymatic reaction. nCounter Human Pan Cancer IO-360 code set + panel standard cat # XT-CSPS-HIO360-12 and Master Kit-NAA-AKIT-012 platform were purchased from nanoString Technology (Seattle, WA). Hybridization was carried in a regular thermal cycler. The hybridized mix was purified using magnetic beads in the nCounter machine in the institute of genome sciences (IGS) University of Maryland Baltimore. Data analysis was carried using IO-360 platform 770 genes code sets using the nSolver 4.0 software basic and advanced custom analysis.

### Statistical Analysis

The distribution of Sema4D and other scale variables was compared between groups of patients using non-parametric statistics, the independent-samples Mann–Whitney U test in case of two groups and the independent-samples Kruskal–Wallis test in case of three or more groups. Associations between scale variables were quantified using Spearman’s rank correlation coefficient, R_s_, and tested against the null hypothesis of R_s_ = 0. Categorical variables were summarized as frequency distributions with indication of the relative frequency and its 95% confidence limits in parenthesis when relevant. All p-values are two-tailed; statistical significance was called for p < 0.05. A Bonferroni correction was applied to adjust for multiple comparisons, whenever relevant. Box-and-whisker plots were generated as a non-parametric representation of the distribution of a scale variable in a group; the sides of the box indicate the 1^st^ and 3^rd^ quartiles of the population distribution with a vertical bar indicating the median. The whiskers extend to 1.5 times the height of the box or, if no case has a value in that range, to the minimum or maximum values. In case of a normally distributed variable, approximately 95% or the data are expected to lie inside the whiskers. Outlying data outside this interval are marked with an asterisk. Binary logistic regression was used for multivariable analysis of patient- and disease-characteristics associated with having elevated sSema4D levels. Statistical analysis of patient engagement, and survey data was conducted using IBM^®^ SPSS^®^ Statistics for Windows, release 24.0.0 (IBM Corp., Armonk, N.Y., USA). nSolver 4.0 analysis uses the student T-test with statistical significance p <0.05.

## Results

### Histological Inflammatory Subtypes Scored Using Sema4D in HNSCC

**Figure 1:**
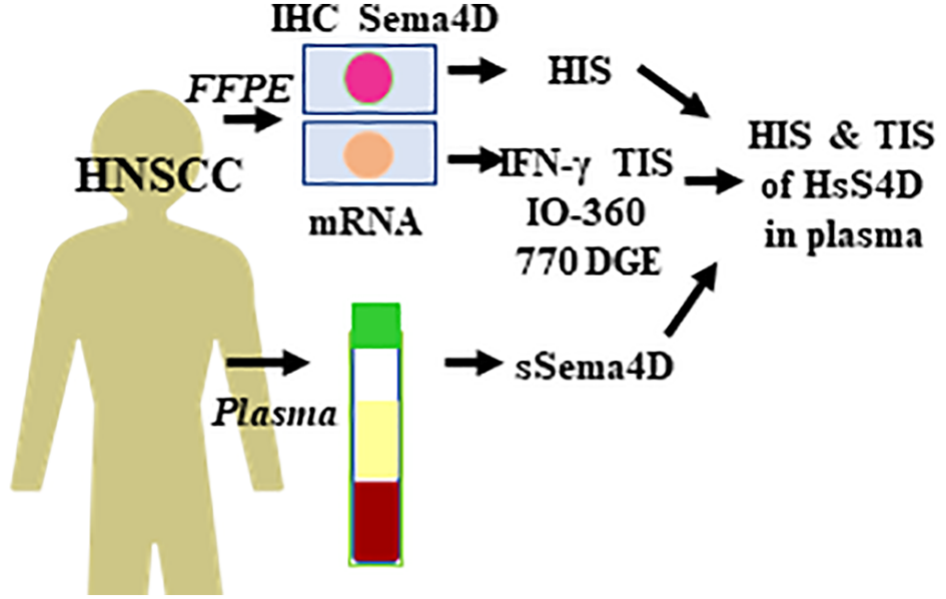
Experimental outline and the workflow. Paired whole excisional tumor tissue and PB collected from 104 HNSCC patients at the same time point to allow for real time analysis of sSema4D in plasma in correlation to HIS and DGE. PB, peripheral blood; sSema4D, soluble Sema4D; IHC, immunohistochemistry; HIS, histological inflammatory subtype; TIS, IFN-*γ* tumor immune signature; DGE, differential gene expression; IO-360, Immuno-Oncologic 770 gene set. HsS4D, high soluble Sema4D in plasma.

**Figure 2:**
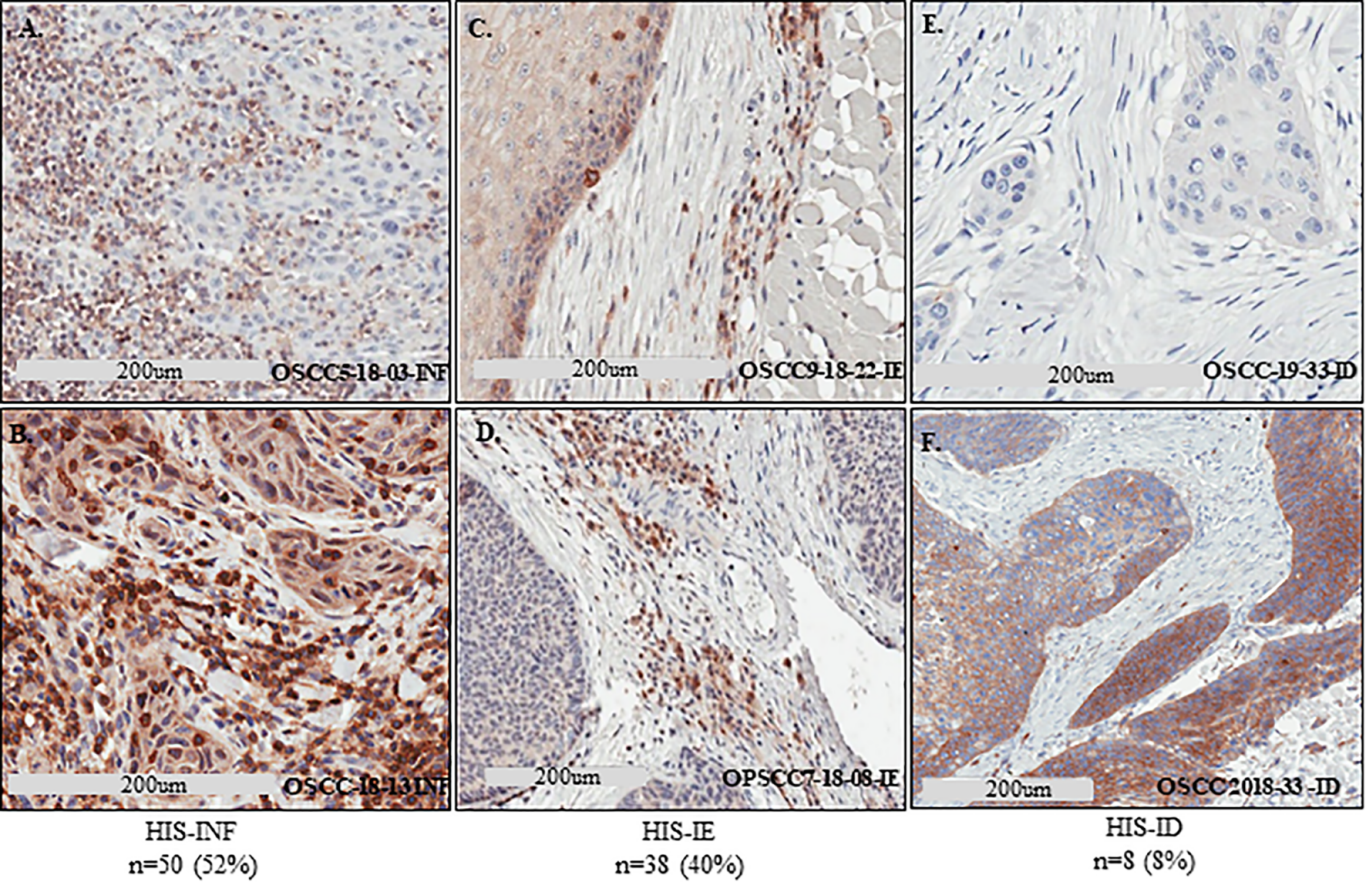
Defining the HIS subtypes in HNSCC using Sema4D. IHC stain of Sema4D in HNSCC tissue. **(A, B).** HIS-INF OSCC tumors demonstrating IC infiltrate into the core of the tumor islands. **(C, D).** HIS-IE in OSCC **(C)** and base of tongue SCC **(D)**, shows IC excluded from tumor island by a rim of PTSF **(C)** or FMX rim **(D)**. **(E, F).** HIS-ID in OSCC showing cold non-inflamed fibrotic dense stroma deserted of IC. IHC, immunohistochemistry; HIS, histological inflammatory stroma subtype; INF, inflamed; IE, immune excluded; ID, immune desert; FMX, fibromyxoid; PTSF, peri-tumoral stromal fibrosis.

Histological patterns of tumor inflammation were described using immunoscore of T cell infiltration in the tumor microenvironment (7, 11). Sema4D is an immune biomarker that may be informative of the global immune contexture and hence facilitates visualization of all leukocytes in the tumor core and peritumoral stroma (11, 21, 22, 25, 32). To describe the pattern of histological inflammatory subtypes (HIS) in the current HNSCC cohort, we performed IHC to assess Sema4D levels on 104 HNSCC that were treated with surgical excision as the initial line of treatment (**Figure 1**). We examined the Sema4D positive IC infiltrate in the invasive tumor front, peritumoral stroma, and tumor core. Eight cases of oral SCC were excluded due to lack of peritumoral stroma. The patients’ demographics and tumor characteristics are described in **Table 1**. Sema4D showed moderate to strong membranous and cytoplasmic staining of the immune cells (IC). In the tumor cells (TC), Sema4D showed membranous and/or cytoplasmic staining that ranged from negative/weak to moderate/strong staining. The IC infiltration into the tumor islands was observed in 50 tumors (52%) that were scored as inflamed HIS (HIS-INF) (**Figures 2A, B**). IC infiltration mainly at the tumor invasive front or between the tumor islands, but excluded by a thin peri-tumoral fibro-myxoid/fibrous rim, was a discriminatory factor to score as immune excluded (HIS-IE). This was observed in 38 cases (40%) (**Figures 2C, D**). The stroma was almost deserted of IC infiltrate in eight cases (8%) and was scored as histologically immune deserted (HIS-ID) (**Figures 2E, F**).

**Table 1:** HNSCC Patients demographics and correlation with sSema4D in blood.

HNSCC	N (%)104 (100%)	Correlation to sSema4D in plasma p- value
Age, median (IQR)	67 (60, 73)	0.32
Gender		
Females	44 (42%)	0.17
Males	60 (58%)	
Race		
African American	7 (7%)	0.021^$^
Caucasian	88 (84%)	(−) 0.008^ϕ^
Hispanic	2 (2%)	
Asians	7 (7%)	
Location		
Oral and mobile	97 (93%)	0.15
Tongue		
Oropharynx	7 (7%)	
Stage^#^		
CIS	3 (2%)	0.29
I	35 (34%)	
II	11 (10%)	
III	10 (9%)	
IV	45 (45%)	
PATH grade		
CIS/Sup inv	7 (7%)	0.34
well	47 (46%)	
mod	39 (38%)	
poorly	10 (9%)	
HPV		
Yes	4 (4%)	0.046
No	100 (96%)	
Smoking		
Yes	43 (41%)	0.103
No	61(59%)	
Alcohol		0.70
Yes	19 (18%)	
No	85 (82%)	
History of dysplasia or HNSCC		
No history	57 (63%	0.01^‡^
History of Dysplasia	5 (6%)	
History of HNSCC	28 (31%)	

Decimals are rounded to the nearest whole number. ^#^AJCC 8^th^ edition staging system. Only significant p values for the HNSCC racial groups are mentioned. Caucasian versus African American^$^ and Caucasian versus Asian ^ϕ^. HPV +ve: one oropharyngeal and three oral SCC. ^‡^HNSCC patients with history of dysplasia showed less level of sSema4D in plasma compared to other HNSCC groups.

### The Sema4D HIS Validated Using IFN-γ Tumor Immune Signature

**Figure 3:**
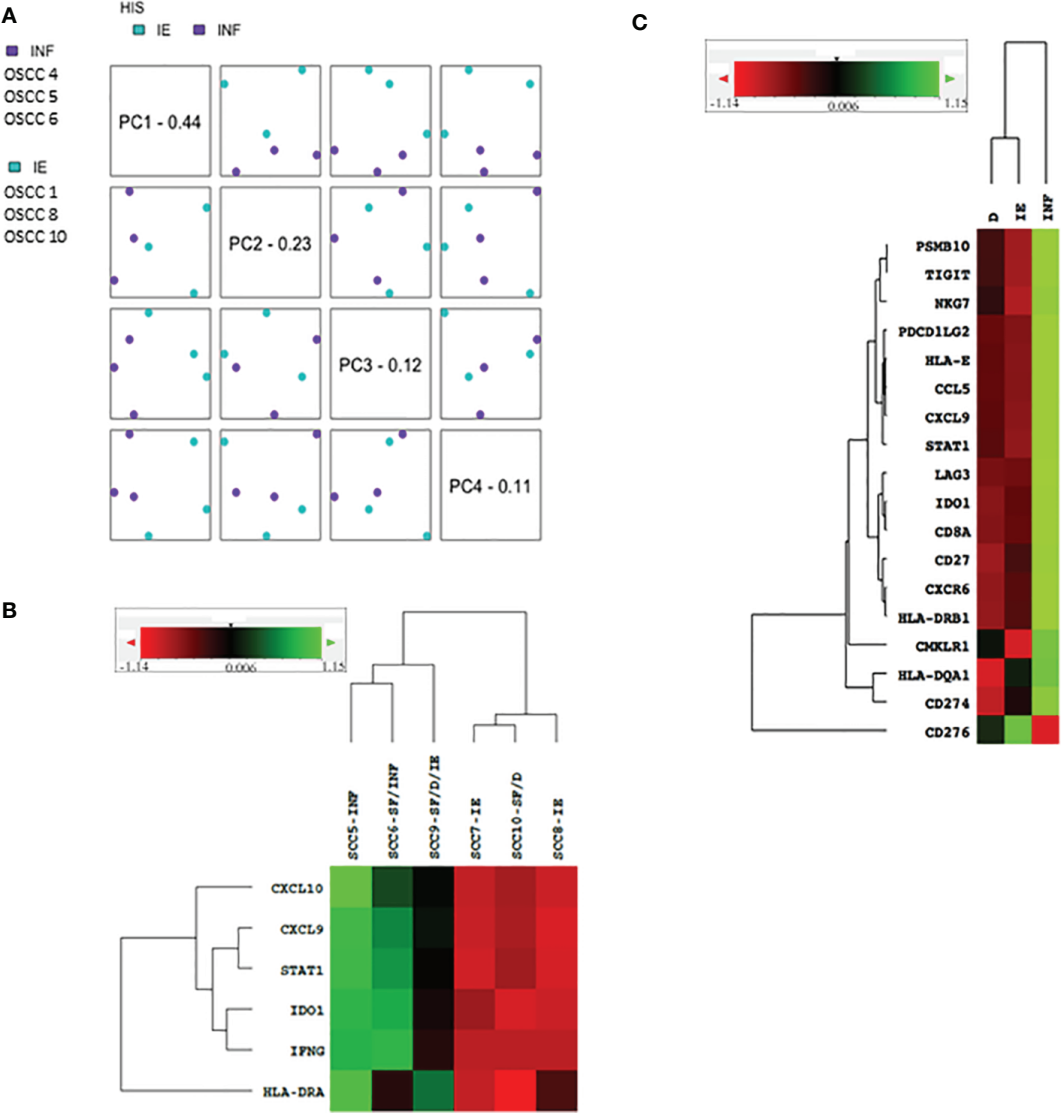
Validation of the Sema4D HIS subtypes using PCA and IFN-*γ* signature on nSolver 4.0 analysis. **(A)** PCA analysis of the HIS-IE and HIS-INF. **(B)** Heat map of the refined six IFN-*γ*-signature in representative HIS tumors. **(C)** Heat map illustrating final IFN-*γ* 18 gene signature between grouped cases of the three HIS subtypes (SCC 5&6 (INF), 7&8 (IE), 9&10 (ID). INF, inflamed; IE, immune excluded; ID, immune deserted; SF, stromal fibrosis. PCA, Principal component analysis. Green is positive; read is negative.

To validate the Sema4D HIS immune score, we tested representative samples using n Solver 4.0, principal component (PC) analysis. Indeed, the HIS-IE and HIS-INF segregate on PC.1 (**Figure 3A**). Then we examined the underlying immune transcriptional profile. We used the T cell inflamed immune signature, composed of active IFN-*γ* signaling, cytotoxic effector, and antigen presentation molecules, and T cell active cytokines that were previously sequentially validated to predict response to standard immunotherapy in HNSCC, as well as other tumor types (15). The refined six gene tumor immune signature (IDO1, CXCL10, CXCL9, HLA-DRA, STAT1, IFNG) was carried on a sample of cases representative of the Sema4D HIS subtypes using basic nSolver analysis (**Figure 3B**). The expanded IFN-*γ* 16 gene signature (CD3D, IDO1,CD3E, CCL5,GZMK, CD2, HLA-DRA, CXCL13, IL2RG, NKG7, HLA-E, CXCR6, LAG3, CXCL10, STAT1, GZMB) was also run on individual samples and on grouped samples. Furthermore, the final validated T cell inflamed 18 gene signature (PSMB10, TIGIT, NKG7, PDCD1LG2, HLA-E,CCL5, CXCL9, STAT1, LAG3, IDO1,CD8A, CD27, CXCR6, HLA-DRB1, CMKLR1, HLA-DQA1, CD274, CD276) (15), was carried out on grouped samples representative of the three HIS subtypes (**Figure 3C**). Interestingly, Sema4D HIS-INF tumors showed a positive IFN-*γ* six gene signature, and the HIS-IE and HIS-ID were negative for the IFN-*γ* six gene signature (**Figure 3B**). The same distribution was observed using the expanded 16 gene (**Supplemental Figure 1**) and the final validated IFN-*γ* 18 gene signature (**Figure 3C**) (15).

### Sema4D in Peripheral Blood of HNSCC Patients

High levels of sSema4D (HsS4D) have been described in chronic inflammatory conditions like osteoarthritis (OA), rheumatoid arthritis (RA), other autoimmune conditions (AI), and allergic reactions; like asthma (A) (38, 39). sSema4D was also previously described in plasma of HNSCC (5). To investigate the potential of sSema4D in peripheral blood as a soluble immune biomarker that can read the level of inflammation in HNSCC patients, we analyzed the level of sSema4D in the plasma of the 104 HNSCC patients compared to the control groups of healthy donors, AI, A, and OA patients (**Supplementary Table 1**). Our data showed that there was no statistically significant difference between sSema4D levels among the AI/A/OA conditions (p = 0.07). The level of sSema4D was highest within the Collagenous AI (Col AI) group (p = 0.011), and in the RA group compared to other Col AI diseases (p = 0.012) (**Supplementary Figure 2**).

sSema4D levels were significantly higher in the AI/A/OA group compared to HNSCC (p = 0.003, adj p = 0.009) and healthy donors (p < 0.001, adj p= 0.001). There was no statistically significant difference observed between sSema4D level in healthy donors and HNSCC (p = 0.051, adjust p = 0.152). However, 75% of HNSCC cases had higher sSema4D levels in plasma than the median of the healthy donors (above 83 ng/ml). There was no statistically significant difference observed between sSema4D levels in plasma and other clinical or demographic characteristics including smoking or alcohol drinking (**Table 1**). The HPV +ve cases and various racial groups were limited in number, which prevented conclusive results related to these variables in the current HNSCC cohort.

### sSema4D in Plasma Reads the Underlying HIS and Immuno-Oncologic Signature in HNSCC

**Figure 4:**
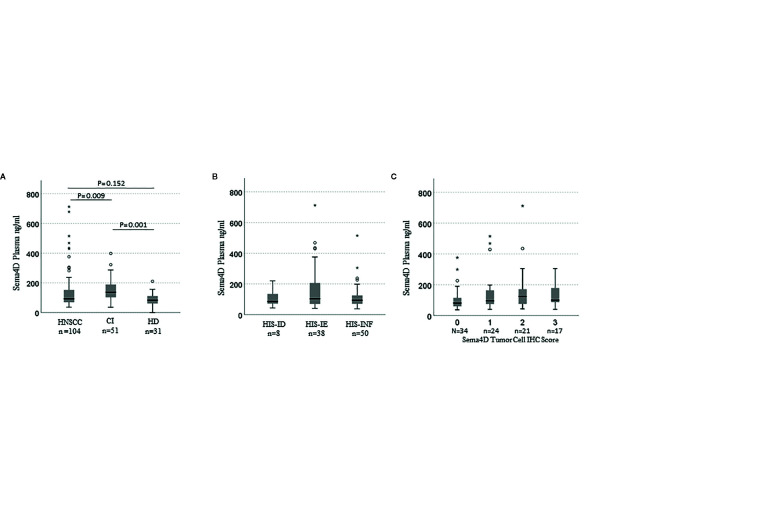
sSema4D in plasma correlates with HIS-IE and Sema4D +ve TC in real time. Box and whisker plots illustrating independent samples Kruskal–Wallis test of **(A)** sSema4D level in plasma in HNSCC, CI (AI/A/OA) and HD. **(B)** The sSema4D in relation to the HIS subtypes (INF, IE, ID). **(C)** sSema4D levels correlate with Sema4D in TC. TC, tumor cell; HIS, histological inflammatory subtype; INF, inflamed; IE, immune excluded; ID, immune deserted. HD, healthy donors; CI, chronic inflammation; AI/A/OA, autoimmune; allergy and osteoarthritis. The circles and asteriks represent samples with higher level brackets.

To investigate the potential of sSema4D as a soluble immune biomarker that can read the underlying tumor inflammatory stromal subtype, we analyzed the level of sSema4D in plasma with the paired tumor HIS subtype. The paired plasma and tumor tissue were collected at the same time point to allow for real time analysis (**Figure 1**). We defined a patient as having elevated levels of sSema4D (HsS4D) if the value exceeded 155 ng/ml, the 95th percentile of the levels measured in healthy donors. Using this definition, 25.0% (95% CI, 16.7–34.9%) of patients with HNSCC presented with elevated Sema4D (**Figure 4A**) (**Supplementary Table 2**). Our data showed a statistically significant association between HIS and HsS4D in plasma, P = 0.007. This is driven by HIS-IE, where the proportion of cases with HsS4D is 42% with exact binomial confidence limits (26%, 59%) as compared with HIS-INF where 14% (6%, 27%) presented with HsS4D (**Figure 4B**, **Table 2**).

**Table 2:** HsS4D in plasma is associated with HIS-IE.

			Plasma		Total	p-value
			LsS4D	*HsS4D		
HIS	ID		7	1	8	
			87.5%	12.5%	100.0%	
	IE		22	16	38	0.007
			57.9%	42.1%	100.0%	
	INF		43	7	50	
			86.0%	14.0%	100.0%	
	Total		72	24	96	
			75.0%	25.0%	100.0%	

*HsS4D defined as sSema4D in plasma >155 ng/ml, LsS4D, Low sSema4D; ID, immune deserted; IE, immune excluded, INF, Inflamed.

The tumor was scored for the Sema4D and PD-L1 in IC and TC. Interestingly, sSema4D levels in plasma correlated significantly with Sema4D^+ve^ TC (p = 0.018) (**Figure 4C**) and PD-L1^+ve^ IC (p = 0.038). Furthermore, Sema4D ^+ve^ TC also correlated with PD-L1^+ve^ IC (p = 0.031). There was no statistically significant correlation observed between sSema4D and Sema4D in IC, PD-L1 in TC, nor PD-L1 or Sema4D CPS (**Supplementary Table 3**).

We performed a multivariable logistic regression analysis to identify patient-level factors associated with elevated HsS4D in patients with HNSCC. Patient-level factors tested in the model were age, gender, race, stage of disease, lympho-vascular invasion, smoking history, alcohol use, HIS, PD-L1 in IC, and PD-L1 in TC. Among these, HIS was highly statistically significant in the final model, P = 0.0014 (Likelihood ratio test) in a model adjusting for PD-L1 in TC (P = 0.035). The *post-hoc* test showed that most of the contrast related to HIS was between HIS-IE and HIS-INF, with an odds ratio for presenting with elevated sSema4D of 6.3 with 95% CI (2.2, 18.4), P = 0.0007 for patients with HIS-IE tumors. This analysis suggests that the information on HIS conveyed by Sema4D is independent of PD-L1.

We further analyzed the final 18 gene IFN-*γ* signature in a sample of 10 cases in relation to the level of sSema4D in plasma, using basic nSolver 4.0 analysis. The 10 cases were selected to include replicates of the HIS patterns (INF *versus* IE &/or ID), and Sema4D +ve and −ve tumor cells. They were also selected to include replicates of Sema4D/PD-L1 co-positive tumor cells *versus* Sema4D-ve/PD-L1+ve tumor cells, as previously characterized (32) (**Supplementary Figure 3**). The reference cutoff value >155 ng/ml for high sSema4D (HsS4D) in plasma was used. Four cases that were scored as HIS-IE or HIS-ID revealed HsS4D in plasma and clustered as negative IFN-*γ* immune signature. On the other hand, two cases of HIS-INF were LsS4D in plasma and clustered as positive IFN-*γ* signature. The remaining four cases represented a gray zone of IFN-*γ* expression, two of which were HIS-IE with a group of downregulated IFN-*γ* genes, and HsS4D in plasma that clustered more towards the positive IFN-*γ* signature. One case of HIS-IE with considerable number of downregulated IFN-*γ* genes was LsS4D in blood and clustered towards IFN-*γ* positive. One case of the HIS-INF, with IFN-*γ* positive signature was HsS4D. Interestingly, this case had a group of downregulated IFN-*γ* genes (**Supplementary Figure 3**).

**Figure 5:**
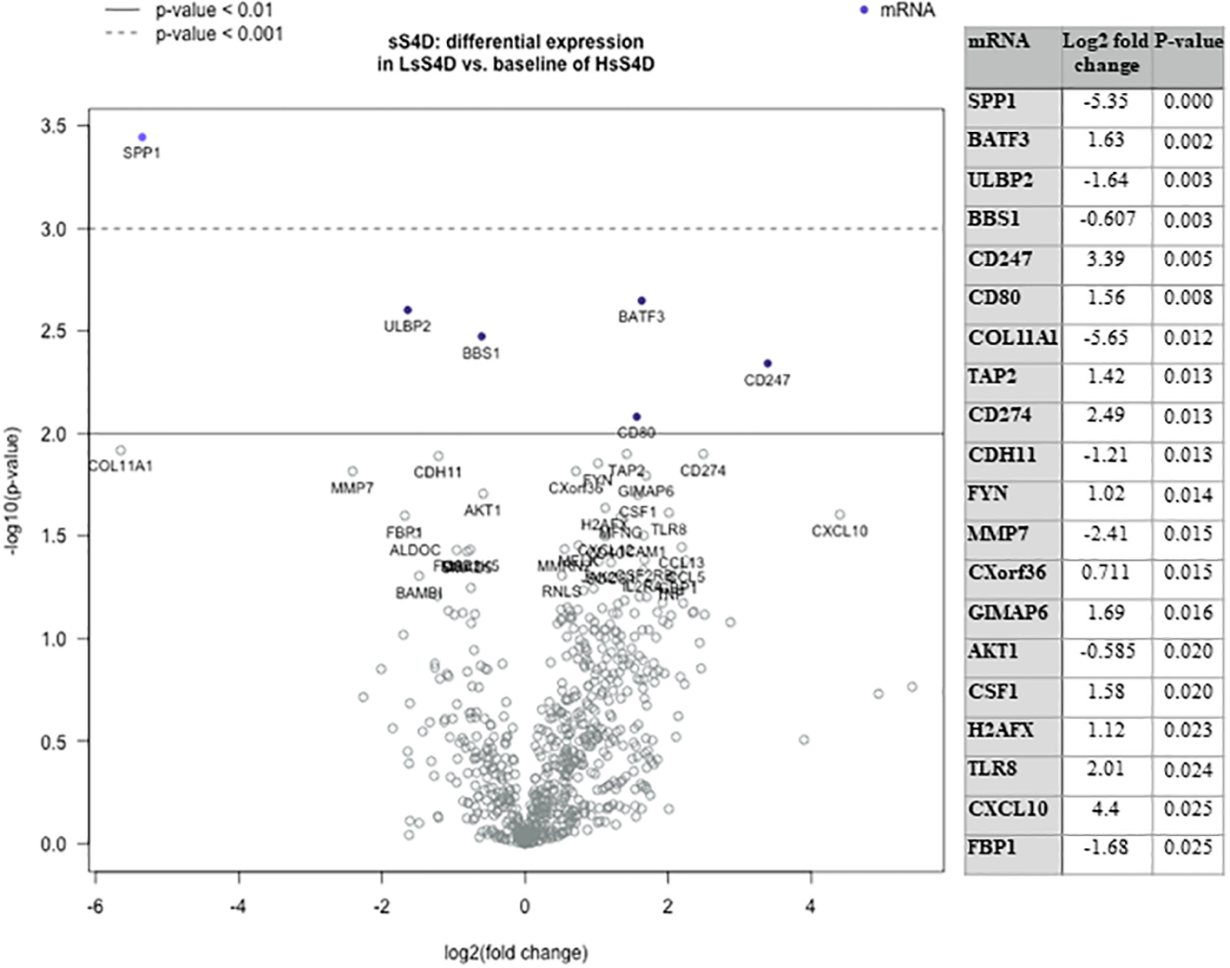
Immuno-oncologic analysis of HNSCC tumor tissue using sS4D as a predictor variable. DGE Volcano plot, presenting linear regression of 770 genes using IO-360, in LsS4D versus baseline of HsS4D. Table presents the top 20 significant DGE genes. Duplicates of LsS4D with IFN-*γ* +ve (SCC05, SCC06), were compared to HsS4D with IFN-*γ* −ve (SCC08 & SCC10) and HsS4D IFN-*γ* +ve (SCC01 and SCC04) (guided by **Supplemental Figure 3**). DGE, differential gene expression; LsS4D, low level of sSema4D in plasma; HsS4D, high level of sSema4D in plasma. P < 0.05 is significant.

**Figure 6:**
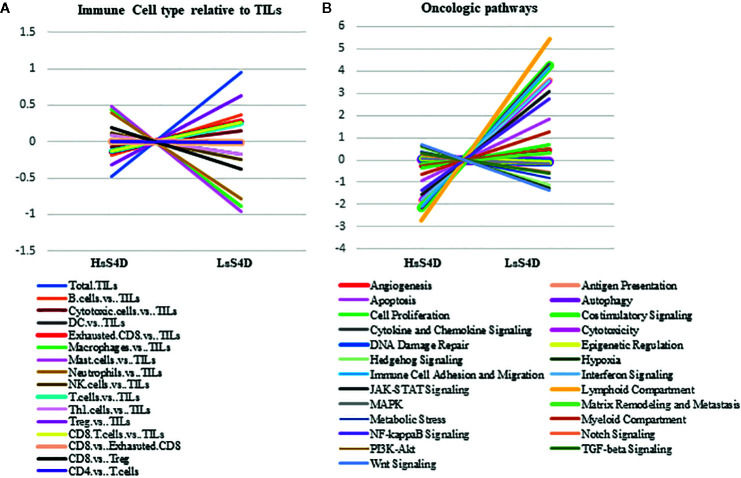
Immune cell type and Immuno-oncologic pathways analysis *versus* sSema4D level in plasma. **(A)** Trend plot of immune cell type relative to total TILs in LsS4D *versus* HsS4D. **(B)** Trend plot analysis of immune-oncologic signaling pathways in LsS4D *versus* HsS4D. The same samples used for **Figure 5** were used. TILs, tumor infiltrating leukocytes; LsS4D, low level of sSema4D in plasma; HsS4D, high level of sSema4D in plasma.

We then investigated the underlying immune-oncologic profile of the HsS4D compared to the LsS4D. We used the IO-360, 770 genes representative of the oncogenic pathways, tumor inflammation and tumor microenvironment. RNA was extracted from mapped FFPE tumor tissue. Using the sSema4D a predictor variable, with a customized, advanced nSolver analysis, differential gene expression of ~40 genes in LsS4D *versus* HsS4D was observed (**Figure 5**). SPP1, ULBP2, COL11A1, and MMP7 were significantly upregulated in HsS4D compared to the LsS4D. The T cell inflamed tumor biomarker CD274 (PD-L1) was significantly upregulated in the LsS4D in addition to BATF3, CD247 (CD3*ζ*), CD80 among others (**Figure 5**). The distribution of the IC type demonstrated total tumor infiltrating leukocytes (TILs) to predominate in LsS4D tumors (**Figure 6A**). Analysis of the IC type relative to total TILs, showed Tregs to be the most numerous followed by B cells, exhausted T cells, then cytotoxic T cells in the LsS4D. Cytotoxic T cells were low in the LsS4D but were still higher than in HsS4D samples. Interestingly, the HsS4D had more mast cells, macrophages, and neutrophils in the TILs (**Figure 6A**). A trend plot of the immune-oncologic signaling pathways showed that the highest was the lymphoid component, followed by costimulatory signaling, cytokines/chemokines, immune cell adhesion and migration pathways. IFN-*γ*, antigen presentation, and cytotoxicity pathways were also elevated. JAK-STAT and NF kappa B pathways were also among the upregulated pathways in the LsS4D compared to the HsS4D. In the HsS4D the Hypoxia and the Wnt signaling pathways were the most upregulated, followed by the Hedgehog pathway, metabolic stress, and TGF-*β*1 signaling (**Figure 6B**). Taken together, these findings suggest that HsS4D in plasma reads the underlying non-inflamed tumor, hypoxic and metabolically stressed microenvironment.

**Figure 7:**
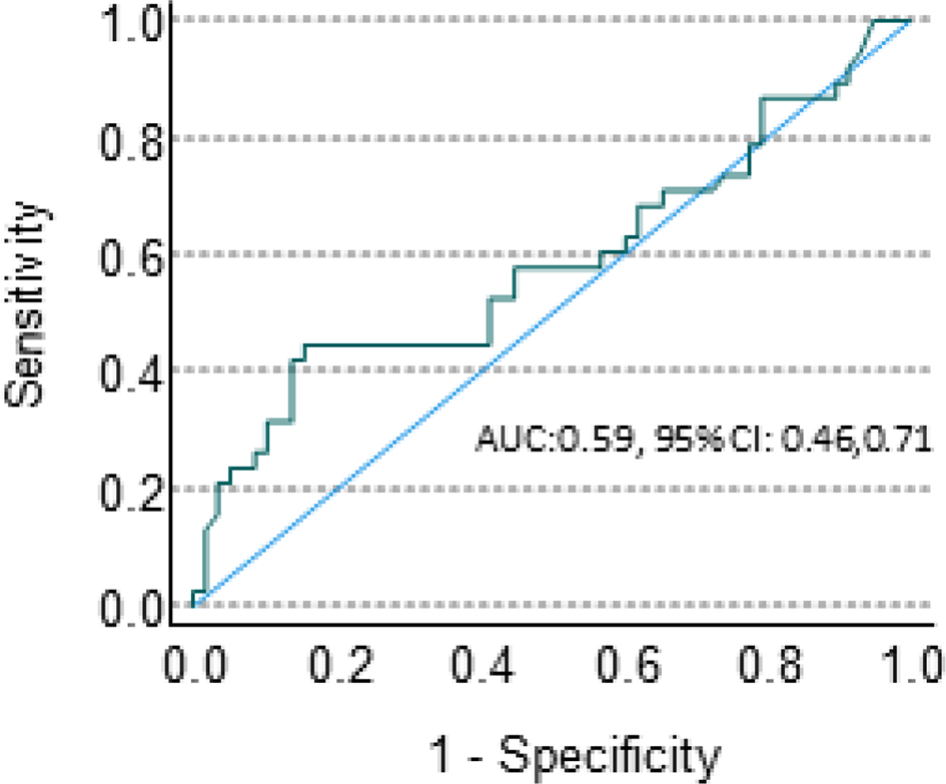
sSema4D ELISA blood assay in HNSCC. ROC curve of the specificity and sensitivity of the HsS4D as a predictive marker of HIS-IE.

This biological association between Sema4D and HIS-IE raises the question whether Sema4D is of potential use as a predictive biomarker for the underlying tumor inflammatory stromal subtype? To this end, we performed a ROC curve analysis. The area under the ROC curve for Sema4D as a predictor of HIS-IE was 0.59 with 95% CI (0.46, 0.71). This was not statistically significantly different from the null hypothesis of AUC = 0.5, P = 0.17. The sensitivity of elevated HsS4D for predicting HIS-IE was 42% with 95% CI (26%, 59%), and the specificity was 86% with 95% CI (74%, 73%) (**Figure 7**).

## Discussion

Here we used Sema4D staining of the immune cells in the tumor microenvironment to score the three patterns of histological inflammatory subtype (INF, IE, ID) (9, 10). It is worth mentioning that scoring of tumor inflammation using Sema4D has not been previously reported. Interestingly, the percentage of INF/immune high *versus* IE/immune low using Sema4D scoring in the current cohort (**Figure 2**) is in concordance with the previously reported immunoscore using CD3/CD8 in HNSCC (7, 40, 41).

Biologically, our findings imply that the HsS4D in HNSCC can modulate a fibrotic type of tumor stromal inflammation similar to the chronic collagenous inflammation in RA and SLE. It can promote immune mediated reactions in cancer patients characterized by a stromal phenotype that acts as an immune exclusion barrier.

The current work demonstrates that a statistically significant association between HIS and HsS4D in plasma, was driven by HIS-IE (P = 0.007). The nanoString analysis of a sample of the HsS4D-paired tissues was mostly IFN-*γ* signature negative, high in the myeloid component, in SPP1, and COLL11A gene expression, Wnt, hypoxia and TGF-*β* signaling (**Figures 5** and **6**). Interestingly, a recent study based on TCGA and GEO data set analysis in HNSCC showed that COL11A1, TGF-β and SPP1 were among the highest scored and selected hub genes. High SPP1 and high TGF-*β* expressing HNSCC were associated with a lower overall survival rate than the low expressing tumors (42).

LsS4D patients had more HIS-INF tumor pairs that were positive for IFN-*γ* signatures with high lymphoid compartments and costimulatory signals. The Tregs predominated the lymphoid compartment in the LsS4D paired tissue. Interestingly, high Tregs in the tumor invasive margin and core were reported to correlate with better survival in HNSCC independent of HPV status (43). In addition, a recent study showed that the proximity of Tregs to CD3/CD8 cells can be a more precise estimate for overall survival of patients with HNSCC rather than summative assessment (8).

Previous studies showed that inhibition of Sema4D in an *in vivo* tumor model facilitated IC infiltration into the tumors and decreased the MDSC component (44, 45). Humanized anti-Sema4D antibody is currently under investigation in cancer and autoimmune neurogenic disorders. It is well tolerated, and the immune cell levels at baseline and progression-free survival were consistent with an immune-mediated mechanism of action (46, 47). Future studies to investigate the level of sSemaD in plasma in response to Sema4D inhibitory antibody and in combination with standard immunotherapy would be informative. The current work suggests that Sema4D level in the blood should be maintained within the healthy donor range to keep the physiologic homeostasis that Sema4D regulates in the immune and the nervous system (20, 25).

Our current cohort is mainly HPV-ve HNSCC of the oral and mobile tongue (**Table 1**), treated with surgery as the initial line of treatment. It is mainly immunotherapy naïve and accordingly provides basal level of the immune biomarker sSema4D in peripheral blood. Although the high level of sSema4D in plasma of AI/A/OA is a limitation to the current technology, none of the patients included in this study had a medical history of any of these conditions. The level of sSema4D in HPV +ve patients, oropharyngeal SCC, as well as patients with previous chemo or radiotherapy, and terminal stage malignancy has yet to be investigated.

Our data suggest that the Sema4D +ve TC can be the source of the sSema4D in blood (**Figures 4C**, **Table 2**), but does not rule out the production of sSema4D in blood by activated circulating immune cells. Intriguingly, cancer patients can demonstrate both pro-inflammatory and anti-inflammatory response, as the patient’s immunity at the tumor tissue level and in the circulation is altered at different stages of tumor development. Further investigations to demonstrate the direct evidence of the source of HsS4D in plasma of HNSCC patients and its correlation with disease progression and patient survival are warranted. This can include future comparative analysis of pre-operative *versus* post-operative levels of sSema4D in plasma of HNSCC.

**Figure 8:**
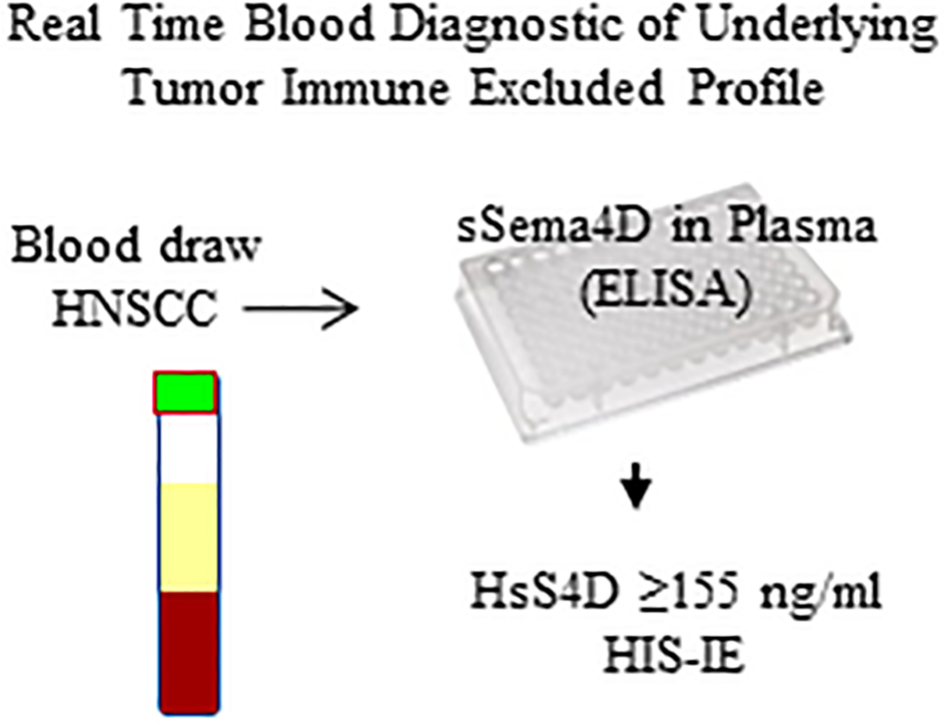
Diagram of the sSema4D ELISA blood assay in HNSCC. HsS4D, High soluble Sema4D; HIS-IE, Histological immune excluded phenotype.

The current work suggests that HNSCC with elevated sSema4D could be a distinct phenotype. The observed associations raise the hypothesis that changes in sSema4D can monitor the underlying dynamics of tumor and stromal inflammation in real time. This would be the topic of a subsequent, larger study (**Figure 8**). Characterization of the differential immune oncologic profile and the histological pattern of inflammation in relation to the soluble immune biomarker Sema4D can provide a translational aspect that can further enhance our understanding and stratification of HNSCC patients.

## Data Availability Statement

The original contributions presented in the study are publicly available. This data can be found here: https://www.ncbi.nlm.nih.gov/geo/query/acc.cgi?acc=GSE163131.

## Ethics Statement

The studies involving human participants were reviewed and approved by the University of Maryland Baltimore Institutional review board, Human Research Protection Program. The patients/participants provided their written informed consent to participate in this study.

## Author Contributions

RY generated the research hypothesis, the experimental design, carried the tumor tissue mapping for RNA extraction, carried the Sema4D immunoscore and the IHC scoring, oversaw the ELISA assay, the RNA extraction, carried the basic and custom analysis on nSolver, and wrote the manuscript. IG ran all the ELISA, for the HNSCC and control plasma samples, and contributed to the immunoscore of the Sema4D IHC. ME and RC ran all the PD-L1 IHC and contributed to the scoring. ME scanned all the Sema4D and PD-L1 slides using aperio scanscope for digital analysis. VT assisted in the IHC. JP contributed to the immunoscore. DD, RO, and JL contributed to the HNSCC clinical diagnosis and patient selection. JL collected all relevant clinical, pathological, and demographic parameters of the patients. TW contributed to the direct ELISA assay design. ED and LT contributed to the data analysis. SB carried out the statistical analysis of the Sema4D level in plasma in correlation to all clinical, demographics, and histological findings and generated the graphs. All authors contributed to the article and approved the submitted version.

## Funding

The current work was funded by the Maryland Technology Development Corporation (TEDCO) MII Phase I (PID 0719-012, UMB-Younis). The Institute for Clinical and Translational Research, University of Maryland Baltimore (150-Younis-ICTR), Angel research fund to the Department of Oncology and Diagnostic Sciences, UMSOD.

## Conflict of Interest

TW is CEO of WebbCures, LLC, co-founder of Screen Therapeutics and serves as an advisor for Immunaccel Labs, LLC.

The remaining authors declare that the research was conducted in the absence of any commercial or financial relationships that could be construed as a potential conflict of interest.

## Supplementary Material

Supplementary Figure 1 Heat map analysis of the HIS subtypes using expanded IFN-*γ* signature. **(A)** Heat map illustrating individual tumors using IFN-*γ* expanded 16 genes signature. **(B)** Heat map of expanded IFN-*γ* 16 genes signature between grouped cases of the three HIS subtypes (SCC 5&6 (INF), 7&8 (IE), 9&10 (ID). INF, inflamed; IE, immune excluded; ID, immune deserted.

Supplementary Figure 2 sSema4D in plasma of AI/A/OA. Box and whisker plot Independent-Samples Kruskal–Wallis test illustrating **(A)** sSema4D level in plasma of AI/A/CI. **(B)** sSema4D in RA *versus* other Col AI conditions. AI, autoimmune diseases; A, Asthma; CI, chronic inflammation (osteoarthritis); other Col AI, other collagenous AI; HD, healthy donors.

Supplementary Figure 3 HsS4D in plasma of HNSCC is associated with negative IFN-*γ* signature. Tumor tissue heat map for the IFN-*γ* 18 gene immune signature with corresponding HIS scoring and sSema4D levels in plasma. The 10 samples were mainly selected based on the HIS, and Sema4D in TC. HIS, histological inflammatory subtype; INF, inflamed; IE, immune excluded; ID, desert; HsS4D, high sSema4D in plasma; LsS4D, Low sSema4D in plasma; TC, tumor cell; Green, positive; Red, negative.

Supplementary Table 1 Descriptive analysis of the AI/A/CI controls for sSema4D in plasma. Collagenous AI conditions: RA, L, SC. Non-collagenous AI conditions: MS, Crohn’s, Sarcoidosis and MG. Three allergy patients had osteoarthritis and one RA had osteoarthritis.

Supplementary Table 2 Descriptive analysis of age, race, and sSema4D in plasma of HNSCC patients, AI/A/OA and HD.

Supplementary Table 3 Sema4D and PD-L1 in tumor cell and immune cell in relation to sSema4D in plasma. Decimals are rounded to the nearest whole. 0; negative, 1; weak, 2; positive, 3; strongly positive. Extent of INF; extent of inflammatory cell present independent of the pattern (INF or IE).
