# Supplementary figures and images for: Soluble Sema4D in Plasma of Head and Neck Squamous Cell Carcinoma Patients Is Associated With Underlying Non-Inflamed Tumor Profile
Source: Front Immunol. 2021 Mar 11;12:596646. doi: 10.3389/fimmu.2021.596646 (PMC7991916; doi:10.3389/fimmu.2021.596646)

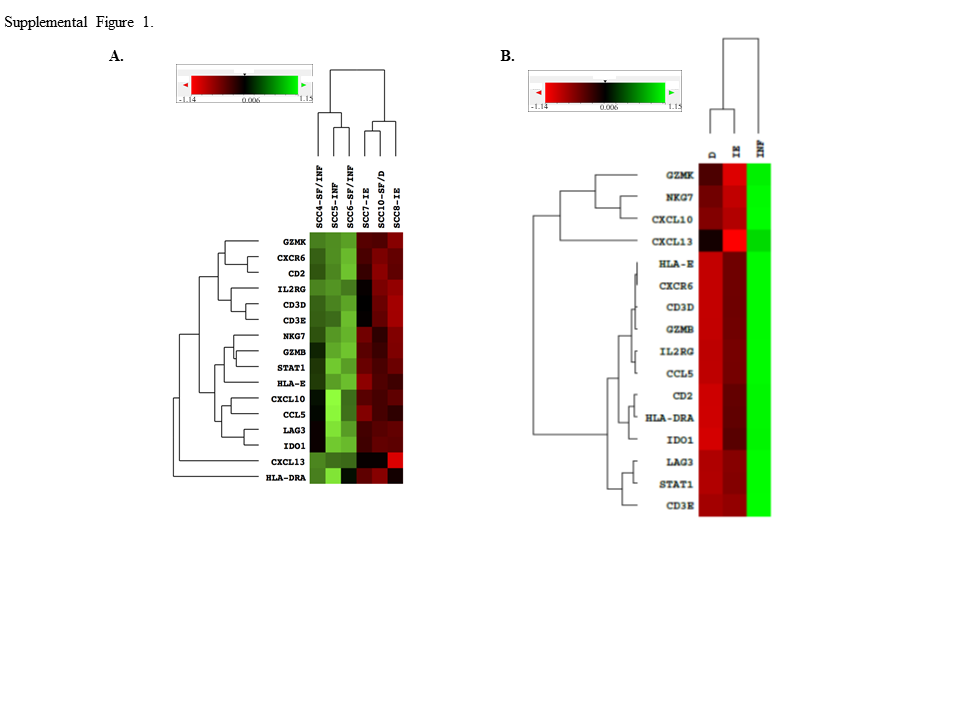

Supplement: Supplementary Figure 1 — Heat map analysis of the HIS subtypes using expanded IFN-γ signature. (A) Heat map illustrating individual tumors using IFN-γ expanded 16 genes signature. (B) Heat map of expanded IFN-γ 16 genes signature between grouped cases of the three HIS subtypes (SCC 5&6 (INF), 7&8 (IE), 9&10 (ID). INF, inflamed; IE, immune excluded; ID, immune deserted. [file Image_1.tif]

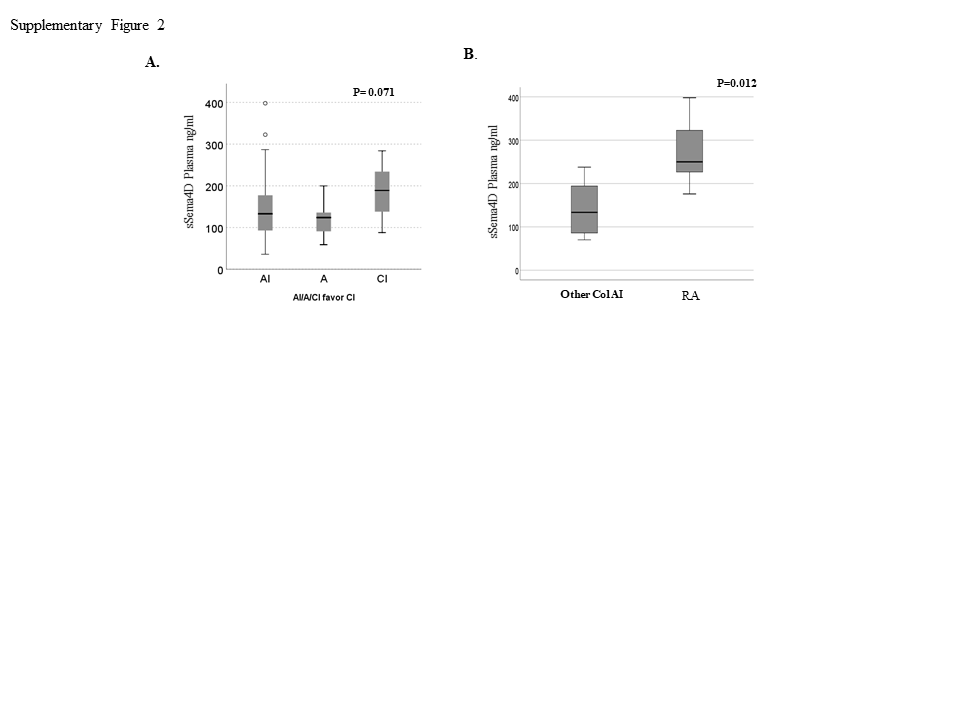

Supplement: Supplementary Figure 2 — sSema4D in plasma of AI/A/OA. Box and whisker plot Independent-Samples Kruskal–Wallis test illustrating (A) sSema4D level in plasma of AI/A/CI. (B) sSema4D in RA versus other Col AI conditions. AI, autoimmune diseases; A, Asthma; CI, chronic inflammation (osteoarthritis); other Col AI, other collagenous AI; HD, healthy donors. [file Image_2.tif]

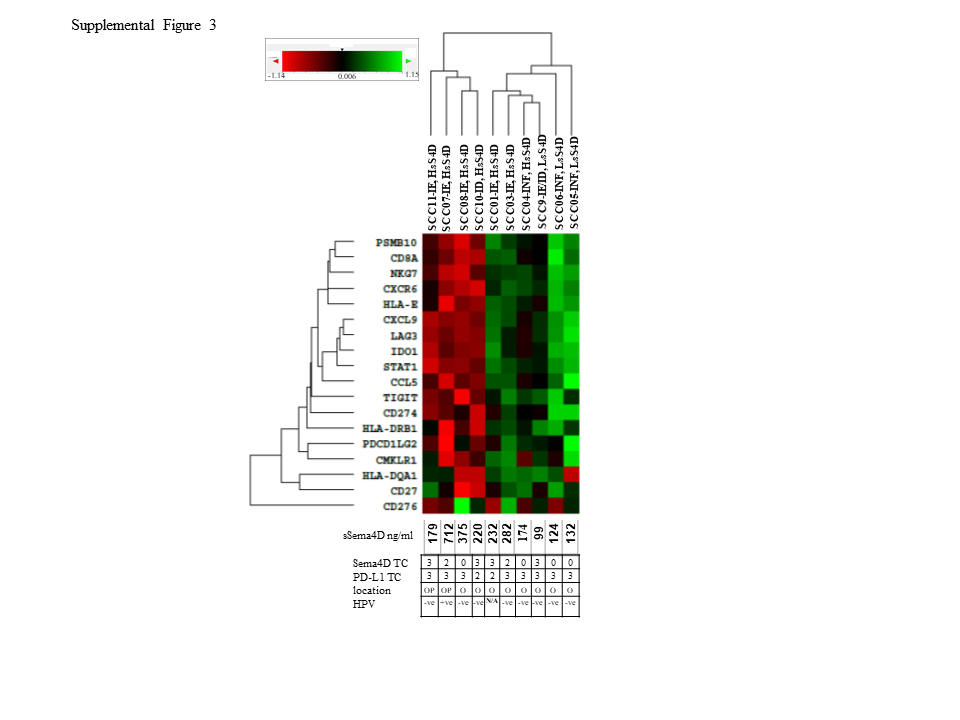

Supplement: Supplementary Figure 3 — HsS4D in plasma of HNSCC is associated with negative IFN-γ signature. Tumor tissue heat map for the IFN-γ 18 gene immune signature with corresponding HIS scoring and sSema4D levels in plasma. The 10 samples were mainly selected based on the HIS, and Sema4D in TC. HIS, histological inflammatory subtype; INF, inflamed; IE, immune excluded; ID, desert; HsS4D, high sSema4D in plasma; LsS4D, Low sSema4D in plasma; TC, tumor cell; Green, positive; Red, negative. [file Image_3.tif]
