# Supplementary material for: Soluble Sema4D in Plasma of Head and Neck Squamous Cell Carcinoma Patients Is Associated With Underlying Non-Inflamed Tumor Profile
Source: Front Immunol. 2021 Mar 11;12:596646. doi: 10.3389/fimmu.2021.596646 (PMC7991916; doi:10.3389/fimmu.2021.596646)
Supplement: Supplementary Table 1 — Descriptive analysis of the AI/A/CI controls for sSema4D in plasma. Collagenous AI conditions: RA, L, SC. Non-collagenous AI conditions: MS, Crohn’s, Sarcoidosis and MG. Three allergy patients had osteoarthritis and one RA had osteoarthritis. [file Table_1.pdf]

Supplemental Table 1: Descriptive analysis of the AI/A/CI controls for sSema4D in plasma.

**Control Groups**		**Number**	**sSema4D in Plasma Range (Average)**
Autoimmune (AI) (31 cases)	Rheumatoid Arthritis (RA)	5	176-398 (274)
	Lupus (L)	5	84-238 (162.8)
	Scleroderma (SC)	1	70
	L, RA	1	88
	Multiple sclerosis (MS)	6	59-287 (142.3)
	Crohn's	7	53-141 (102.4)
	Sarcoidosis	5	36-179 (101.8)
	Myasthenia gravis (MG)	1	114
Allergy (10 cases)	Asthma	13	59-240 (138.2)
Chronic Inflammation (10 cases)	Osteoarthritis (OA)	10	122-240 (182.4)

Collagenous AI conditions: RA, L, SC. Non collagenous AI conditions: MS, Crohn's, Sarcoidosis and MG. 3 allergy patients had osteoarthritis and 1 RA had osteoarthritis.

Supplemental Table 2: Descriptive analysis of age, race, and sSema4D in plasma of HNSCC patients, AI/A/OA and HD.

	HNSCC	AI/A/OA	HD
Number of cases	104	51	31
sSema4D in PLASMA			
Minimum	36	36	0
25% Percentile	69.25	102	59
Median	93	136	83
75% Percentile	152.8	198	113
Maximum	712	398	211
Range	676	362	211
Mean	136.0	150.1	86.3
Std. Deviation	122.7	75.10	46.0
Std. Error of Mean	12.03	10.5	8.3
Lower -upper 95% CI of mean	112-160	129-171	69-103
95% CI of median	96.10%	95.11%	97.06%
Lower-upper confidence limit	87-110	122-152	65-109
AGE			
Minimum	21	19	18
25% Percentile	60	41	25
Median	66.50	53	31
75% Percentile	73	63	45
Maximum	93	83	66
Range	72	64	48
Mean	66.5	52.2	35.7
Std. Deviation	11.4	15.	12.6
Std. Error of Mean	1.1	2.1	2.3
95% CI of median	*96.10%*	*95.11%*	*97.06%*
Lower-upper confidence limit	*62-69*	*50*	*26*
RACE			
AA	7	21	9
Caucasian	88	26	15
Hispanic	2	0	7
Asian	7	2	0
AI	0	0	0
Others	0	1	0
AA, AI, Caucasian	0	1	0
GENDER			
Female	44	36	14
Male	60	15	17

Supplemental Table 3. Sema4D and PD-L1 in tumor cell and immune cell in relation to sSema4D in plasma.

SEMA4D IN TUMOR	N (%)	CORRELATION TO sSEMA4D P-VALUE
**SEMA4D TC**		
**0**	34 (35%)	
**1**	24 (25%)	0.018
**2**	21 (22%)	
**3**	17 (18%)	
**TOTAL**	96 (100%)	
**SEMA4D IC**		
**0**	6 (6.3 %)	
**1**	5 (5.2 %)	0.243
**2**	11 (11.5%)	
**3**	74 (77.1%)	
**TOTAL**	96 (100%)	
**SEMA4D CPS**		
**0**	3 (3.1%)	
**1**	4 (4.2%)	0.214
**2**	10 (10.4%)	
**3**	79 (82.3%)	
	96 (100%)	
**PD-L1 TC**		
**0**	8 (8.4)	
**1**	7 (7.4)	0.249
**2**	12 (12.6)	
**3**	68 (71.6)	
	95 (100%)	
**PD-L1 IC**		
**0**	12 (13%)	
**1**	3 (3%)	0.038
**2**	7 (7%)	
**3**	74 (77%)	
	96 (100%)	
**PD-L1 CPS**		
**0**	5 (5%)	
**1**	0 (1%)	0.241
**2**	3 (3%)	
**3**	88 (92%)	
	96 (100%)	
**EXTENT OF INF**		
**0**	3 (3%)	
**1**	6 (6%)	(-) 0.874
**2**	37 (39%)	
**3**	50 (52%)	
	96(100%)	

Decimals are rounded to the nearest whole. 0; negative, 1; weak, 2; positive, 3; strongly positive. Extent of INF; extent of inflammatory cell present independent of the pattern (INF or IE), TC; tumor cell, IC; immune cell, CPS; compound positive score.
